# Lysyl oxidase engineered lipid nanovesicles for the treatment of triple negative breast cancer

**DOI:** 10.1038/s41598-021-84492-3

**Published:** 2021-03-03

**Authors:** Alessandro De Vita, Chiara Liverani, Roberto Molinaro, Jonathan O. Martinez, Kelly A. Hartman, Chiara Spadazzi, Giacomo Miserocchi, Francesca Taraballi, Michael Evangelopoulos, Federica Pieri, Alberto Bongiovanni, Laura Mercatali, Ennio Tasciotti, Toni Ibrahim

**Affiliations:** 1 Osteoncology and Rare Tumors Center, IRCCS Istituto Romagnolo Per Lo Studio Dei Tumori (IRST) “Dino Amadori”, Meldola, Italy; 2 Department of Cardiovascular Medicine, Brigham and Women’s Hospital, Harvard Medical School, Boston, MA USA; 3 Orthopedics and Sports Medicine, Houston Methodist Hospital, Houston, TX USA; 4 Business Development of Research, IRCCS San Raffaele Hospital, Milan, Italy; 5 Pathology Unit, Morgagni-Pierantoni Hospital, Forlì, Italy; 6 Biotechnology Program, San Raffaele University and IRCCS San Raffaele, Rome, Italy

**Keywords:** Nanomedicine, Nanoscience and technology, Oncology, Cancer

## Abstract

In the field of oncology research, a deeper understanding of tumor biology has shed light on the role of environmental conditions surrounding cancer cells. In this regard, targeting the tumor microenvironment has recently emerged as a new way to access this disease. In this work, a novel extracellular matrix (ECM)-targeting nanotherapeutic was engineered using a lipid-based nanoparticle chemically linked to an inhibitor of the ECM-related enzyme, lysyl oxidase 1 (LOX), that inhibits the crosslinking of elastin and collagen fibers. We demonstrated that, when the conjugated vesicles were loaded with the chemotherapeutic epirubicin, superior inhibition of triple negative breast cancer (TNBC) cell growth was observed both in vitro and in vivo. Moreover, in vivo results displayed prolonged survival, minimal cytotoxicity, and enhanced biocompatibility compared to free epirubicin and epirubicin-loaded nanoparticles. This all-in-one nano-based ECM-targeting chemotherapeutic may provide a key-enabling technology for the treatment of TNBC.

## Introduction

In the landscape of drug delivery systems, a multitude of nanovectors has been developed for cancer treatment. However, targeting the tumor microenvironment has only recently been explored as an option to deliver chemotherapeutics selectively to the tumor site^1,2^. Increasing evidence is demonstrating that the microenvironment plays a key role in tumorigenic events^3–5^. Specifically, the extracellular matrix (ECM) represents a primary component of the tumor microenvironment and its remodeling promotes several tumor processes. Recently, various studies^4,6–8^ attempted to clarify poorly understood mechanisms on how the ECM affects tumor cell proliferation, dissemination, invasion, and metastasis. Therefore, novel strategies implementing specific targeting of the tumor microenvironment could usher in a new generation of therapeutic agents with a favorable impact on tumor therapy.

Within this context, the ECM-associated enzyme lysyl oxidase 1 (LOX) represents a promising candidate for selective drug delivery to tumors. This ECM-remodeling protein is overexpressed in both primary and metastatic lesions of various tumors including breast, pancreas, and bone^4,9–16^. The spectrum of LOX activity is wide and it includes the cross-linking of elastin and collagen fibers^17,18^, the modulation of the structure and stiffness of tumor ECM^19,20^, and the regulation of cell migration and adhesion^21,22^. These findings lead to considerable interest in LOX involvement in tumor pathophysiology and in its potential for improving cancer therapy. Previous studies demonstrated that metastatic growth from breast, prostate, and lung tumors could be slowed or arrested by LOX activity’s inhibition using local or systemic injection of antibodies, pro-peptides, or small molecules^4,23–27^.

Although promising results have been obtained using LOX-directed molecules, accumulation of these molecules in healthy organs has limited their clinical translation due to issues with significant toxicity and adverse side effects^28,29^. Recently, preliminary evidence of functionalizing the surface of poly(lactic-co-glycolic acid) PLGA-nanoparticles with a LOX-blocking antibody demonstrated promising results in suppressing cancer cell growth^30^. However, no studies have been performed to develop a lipid vesicle targeting LOX, combined with chemotherapy-delivery to evaluate its efficacy compared to standard clinical treatment available for breast cancer patients (e.g., epirubicin). Herein, we engineered a lipid-based vesicle functionalized with a LOX antibody and loaded with epirubicin. The goal of this work was to develop a lipid-based vesicle that simultaneously exploits the intrinsic therapeutic activity of targeting LOX in the tumor ECM and selectively concentrates epirubicin at the tumor site, with minimal systemic toxicity. This multifaceted delivery approach could represent a significant breakthrough in the chemotherapy treatment of TNBC, significantly reducing systemic toxicity.

## Results

### Engineered-anti-LOX liposomes designing study

**Figure 1:**
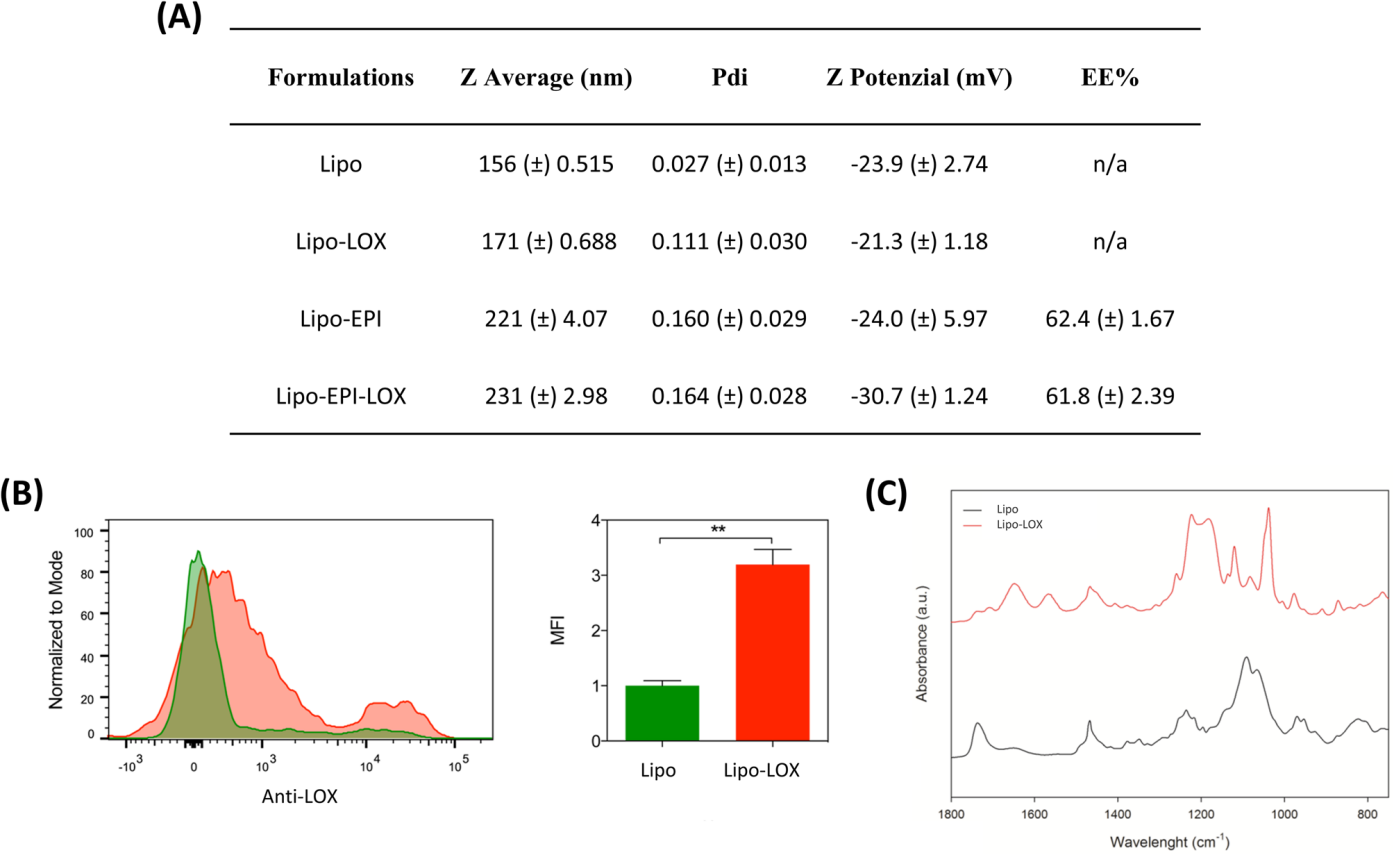
(**A**) Physical characterization of lipid-based nanocarriers. (**B**) Cytometry analysis of Lipo and Lipo-LOX formulations. (**C**) FTIR analysis of Lipo and Lipo-LOX formulations.

We fabricated biocompatible polyethylene glycol (PEG) PEGylated liposomes^31^ (Lipo) through the well-established thin-layer evaporation (TLE) technique as previously reported^32,33^. Liposomes were functionalized with a LOX antibody through conjugation to the carboxyl functional group on the PEG terminus (Lipo-LOX)^34^. To obtain optimal surface coverage, three different anti-LOX to lipid ratios were tested: 1:1000, 1:500, and 1:300. An increase in anti-LOX ratios demonstrated a steady decrease in zeta potential, indicating successful incorporation into the bilayer (Supplementary Fig. 1A). Also, physicochemical characterization of the three formulations demonstrated that anti-LOX incorporation resulted in a reduction in particle size when compared to control liposomes, with no differences observed between the tested anti-LOX ratios (Supplementary Fig. 1B). Increased homogeneity of the formulation was also found, as indicated by the decrease in the polydispersity index (PDI; Supplementary Fig. 1C). Next, flow cytometry analysis was performed to determine the optimal anti-LOX ratio needed to achieve the highest amount of anti-LOX bound to the liposome surface (Supplementary Fig. 1D). The analysis revealed that the 1:500 ratio expressed the highest intensity of the LOX antibody compared to 1:300 and 1:1000. This result could be attributed to the saturation of the carboxyl functional group of liposomes. Based on the higher anti-LOX expression and smaller PDI, all subsequent experiments were carried out using an anti-LOX:lipid ratio of 1:500. Validation of the LOX antibody bound to the liposome surface was confirmed using flow cytometry (Fig. 1B) and Fourier Transform Infrared (FTIR) spectroscopy analysis (Fig. 1C). Classical protein absorption moieties were found in the spectra, amide I (1700–1600 cm^−1^), amide II (1580–1510 cm^−1^), and amide III (1400–1200 cm^−1^), confirming the presence of the LOX protein inside the lipidic structure of nanoparticles. The efficiency of anti-LOX conjugation to liposomes was determined through both a protein analysis and a LOX fluorescent secondary antibody using an anti-LOX calibration curve. The analyses revealed a coating concentration of ~ 20 µg/ml that corresponds to 1 µg of anti-LOX per 1000 µg of lipid components. The concentration of the anti-LOX used was strongly reduced compared to previous work^30^, aiming to prevent in vivo toxicity to obtain a result more translatable to the clinic.

Anthracycline-based regimens represent one of the standard therapeutic options for triple-negative breast cancer (TNBC) patients. Although these molecules achieve a higher pathological response rate (i.e., 30.9–36.4%)^35^ in comparison to other chemotherapies, they suffer from limited tumor targeting and systemic toxicity. For this reason, it is necessary to find strategies to selectively concentrate anthracyclines at the tumor site. Specific ECM tumor-targeting and intrinsic antitumor activity of LOX could enhance the efficacy of standard anthracycline-based regimens by directing accumulation to tumors. Therefore, we designed an anti-LOX engineered drug delivery system to efficiently target and concentrate anthracyclines within the tumor ECM. Anti-LOX-functionalized liposomes (Lipo-LOX) were loaded with the chemotherapeutic, epirubicin (EPI), using previously established loading techniques^36^. This resulted in an encapsulation efficiency of 60% for both Lipo and Lipo-LOX, indicating that anti-LOX surface-bound did not affect drug-loading capacity (Supplementary Fig. 1E). Then, to assess the therapeutic efficacy of anti-LOX and epirubicin combination, four different lipid-based formulations were synthesized: Lipo, Lipo-LOX, epirubicin-loaded liposomes (Lipo-EPI), and epirubicin-loaded anti-LOX liposomes (Lipo-EPI-LOX). The composition and the main physicochemical characteristics of the different preparations were reported in Fig. 1A (Fig. 1A). Notably, the diameters of Lipo and Lipo-LOX were in the range of 156 to 171 nm, respectively, while increased sizes were observed following encapsulation of epirubicin (> 220 nm). Anti-LOX surface-bound led to an increased surface charge (-23 to − 21 mV) while the incorporation of epirubicin resulted in a decreased charge (-30 mV).

### In vitro validation of anti-LOX activity anchored to liposomes

**Figure 2:**
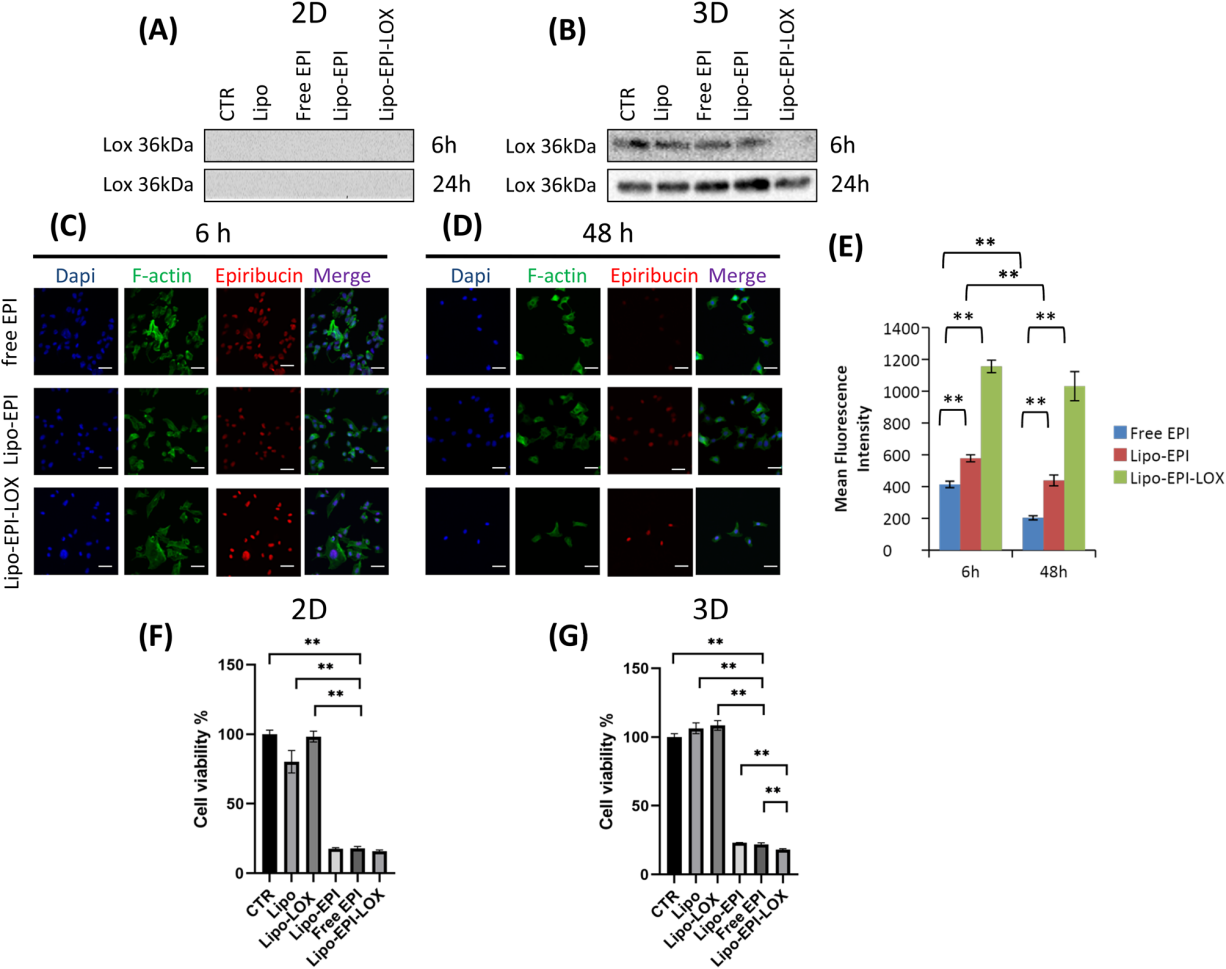
(**A**) Protein analysis of secreted LOX in monolayer cultures not treated and treated with all formulations, full-length blots/gels are presented in Supplementary Fig. 2A. (**B**) Protein analysis of secreted LOX in 3D cultures not treated and treated with all formulations, full-length blots/gels are presented in Supplementary Fig. 2B, C. (**C**) Confocal analysis of 3D cultures exposed to formulations encapsulated with anthracycline after 6 h from the exposure. Nuclei were stained with dapi (blue), actin filaments were stained with phalloidin (green), epirubicin (red) and merge. (**D**) Confocal analysis of 3D cultures exposed to formulations encapsulated with anthracycline after 48 h from the exposure. Scale bar 50 µm (**E**) Mean fluorescence intensity of anthracycline detected after 6 and 48 h. (**F**) Cell viability of tumor cells after treatment with all studied formulations in standard monolayer cultures, negative control is CTR and positive control is LIPO-EPI-LOX. (**G**) Cell viability of tumor cells after treatment with all studied formulations in 3D cultures, negative control is CTR and positive control is LIPO-EPI-LOX. *p < 0.05, **p < 0.01.

Functional LOX antibody on liposomes’ surface was confirmed by assessing the detection’s abrogation of LOX protein by human TNBC cells (i.e., MDA-MB-231) cultured in both standard monolayer (2D) and three dimensional (3D) models. Secreted LOX protein was not detectable in standard 2D culture in both the control and treatment groups at various time points, confirming the need of ECM support for the LOX’s synthesis by MDA MB 231 cells. (Fig. 2A, Supplementary Fig. 2A)*.* However*,* when MDA-MB-231 were cultured on 3D collagen scaffold, secreted LOX was observed after 6 h (Fig. 2B, Supplementary Fig. 2B). Likely, secretion of LOX protein by MDA MB 231 cells in the 3D model could be a cell response to the more aggressive features of the 3D microenvironment and to the direct cell contact with collagen fibers ^9^. To confirm that the synthesis did not affect the bioactivity of anti-LOX, 3D cultures of MDA-MB-231 were exposed to lipid-based vesicles and the secreted free LOX was analyzed. Protein expression results demonstrated that LOX was detectable in Lipo, EPI, and Lipo-EPI, while its expression was initially inhibited by Lipo-EPI-LOX at 6 h before reappearing at 24 h (Fig. 2B Supplementary Fig. 2B, C). These results suggest that liposomes functionalized with anti-LOX retain the bio-activity associated with anti-LOX, confirming the feasibility of implementing an anti-LOX-functionalized system for cancer therapy.

### In vitro assessment of engineered-anti-LOX liposomes for tumor targeting, drug delivery, and cytotoxicity

Functional in vitro assays to investigate the ability of Lipo-EPI-LOX internalization compared to standard treatment groups EPI and Lipo-EPI were performed using MDA-MB-231 cultured in 3D. Confocal analysis demonstrated a significant increase in EPI delivery at both time points (i.e., 6 and 48 h) when encapsulated within Lipo-EPI-LOX particles and compared to free EPI and Lipo-EPI (Fig. 2C–E, EPI vs. LIPO-EPI-LOX at 6 h: *p* < 0.01 and LIPO-EPI vs. LIPO-EPI-LOX at 6 h: *p* < 0.01; EPI vs. LIPO-EPI-LOX at 48 h: *p* < 0.01 and LIPO-EPI vs. LIPO-EPI-LOX at 48 h: *p* < 0.01). These results confirmed the superior in vitro activity achieved by LIPO-EPI-LOX in delivering EPI to TNBC.

Next, the cytotoxic effect of Lipo-EPI-LOX on the viability of MDA-MB-231 cultured in 2D and 3D was assessed (Fig. 2F,G). In 2D, Lipo-EPI-LOX resulted in 85% reduction in TNBC cell viability, Lipo-EPI, and Free EPI displayed similar values (Fig. 2F). In 3D culture systems, Lipo-EPI-LOX displayed a similar reduction in cell viability with more than 80% cell death (*p* < 0.01) with Lipo and Lipo-LOX exhibiting no significant effect on survival and Lipo-EPI and Free EPI showed ˂ 80% of cell death. These results confirmed that the incorporation of anti-LOX did not affect the drug efficacy of EPI or liposomes, enabling Lipo-EPI-LOX to similarly inhibit the proliferation of TNBC cells in comparison to the other EPI controls.

The less reliability of 2D systems, compared to 3D models, could partially explain the current gap between preclinical and clinical data ^9^. For example, our results from 2D were unable to show the effective spectrum of anti-LOX activity (Fig. 2A). Furthermore, we observed differences in MDA-MB-231 cell response to treatment at lower doses between 2 and 3D models (cell viability % in 2D with epirubicin one-quarter plasma peak concentration Lipo-EPI ˂ 20%, EPI ˂ 20%, LIPO-EPI-LOX ˂ 20%; cell viability % in 3D with epirubicin one-quarter plasma peak concentration Lipo-EPI ˃ 50%, EPI ˃ 60%, Lipo-EPI-LOX ˂ 40%, Supplementary Fig. 3, and Supplementary Fig. 4). The similar activity of LIPO-EPI-LOX in 2D cultures was observed by reducing the drug concentration to as low as one-quarter of the plasma peak of anthracyclines (Supplementary Fig. 3C, D), suggesting the breast cancer cells reached drug saturation values. Results obtained in 3D cultures showed an increase in cell viability with all of the tested formulations when the drug concentration was reduced to one-quarter of the plasma peak (Supplementary Fig. 4C). In all experiments, Lipo-EPI-LOX exhibited the highest inhibition in cell viability and achieved the lowest IC_50_ (Supplementary Fig. 4C, D). These results can be explained by the ability of 3D collagen-based scaffold mimicking the in vivo growing conditions of tumors since it incorporates features of the ECM. ECM-macromolecules affect tissue tension, oxygen and nutrients availability, and drug diffusion. For these reasons, the in vivo tumor microenvironment is not uniformly exposed to the same drug concentration. As a result, the development of drug delivery systems capable of selectively convey drugs to tumor lesions would provide new avenues for the treatment of TNBC. Finally, since the mechanical features of tumors and tumor cells are predictive of the disease’s clinical behavior^37^, we investigated the effect of all treatment groups on the stiffness properties of collagen scaffolds. Results showed an average compressive modulus of 67.3 kPa for CTR, 64.28 kPa for Lipo, 61.78 kPa for Lipo-LOX, 64.38 kPa for free EPI, 59.81 kPa for Lipo-EPI, and 55.64 kPa for Lipo-EPI-LOX (Supplementary Fig. 6). In these experiments, only Lipo-EPI-LOX exhibited a significant reduction of scaffold stiffness (*p* ˂ 0.05) compared to CTR. These data support the importance of developing a drug delivery system able to modify the mechanical properties of the tumor microenvironment as a new weapon for the treatment of TNBC.

**Figure 3:**
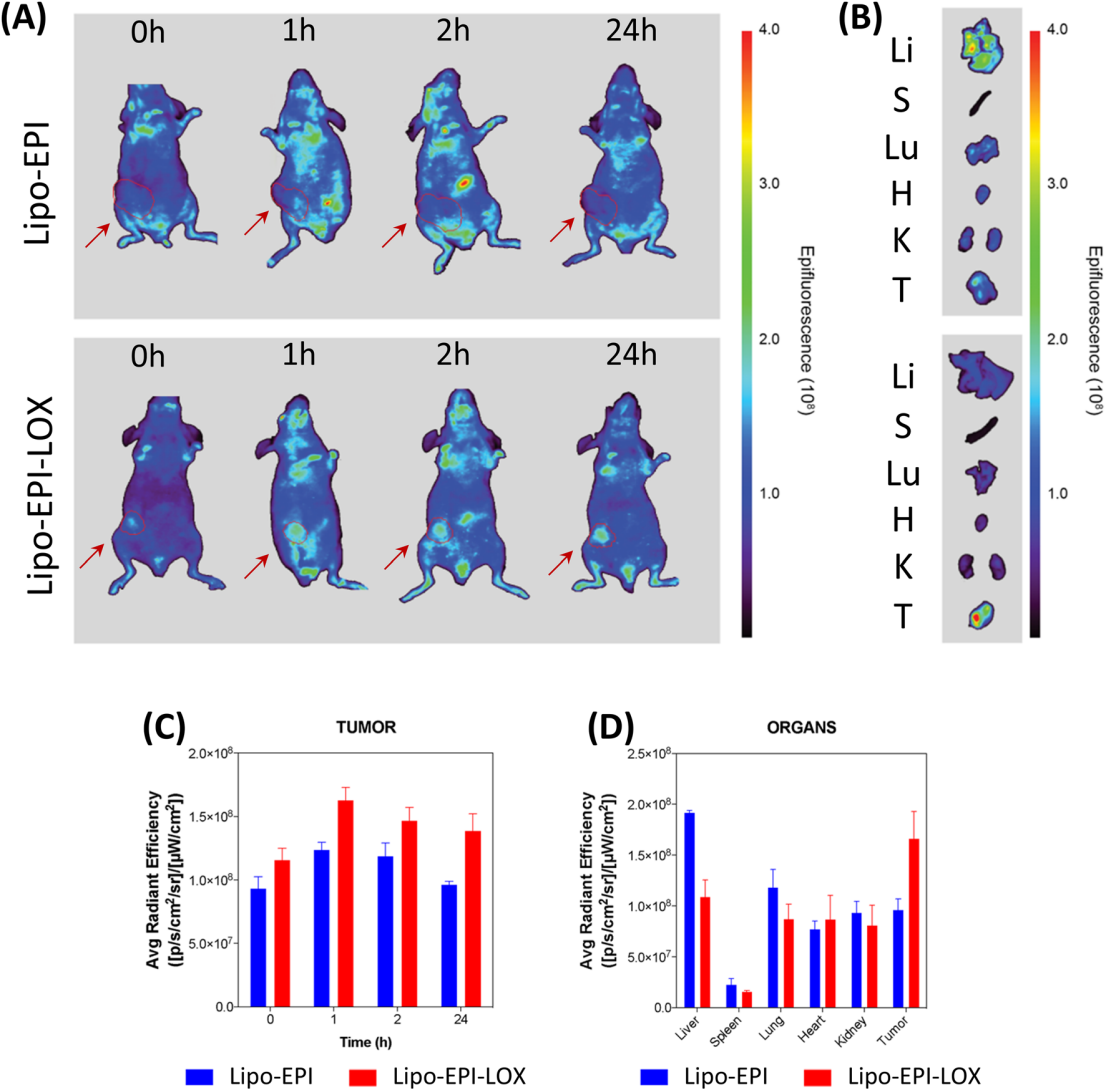
(**A**) In vivo biodistribution analysis of Lipo-EPI and Lipo-EPI-LOX in orthotropic xenograft mouse model of human TNBC at different time points (0, 1, 2 and 24 h post injections). (**B**) bioluminescence and fluorescence analysis of explanted organs (liver, spleen, lungs, heart, kidneys, tumors) to detect EPI localization. (**C**) Lipo-EPI and Lipo-EPI-LOX fluorescent signal quantification in tumor. (**D**) Lipo-EPI and Lipo-EPI-LOX fluorescent signal quantification in explanted organs.

**Figure 4:**
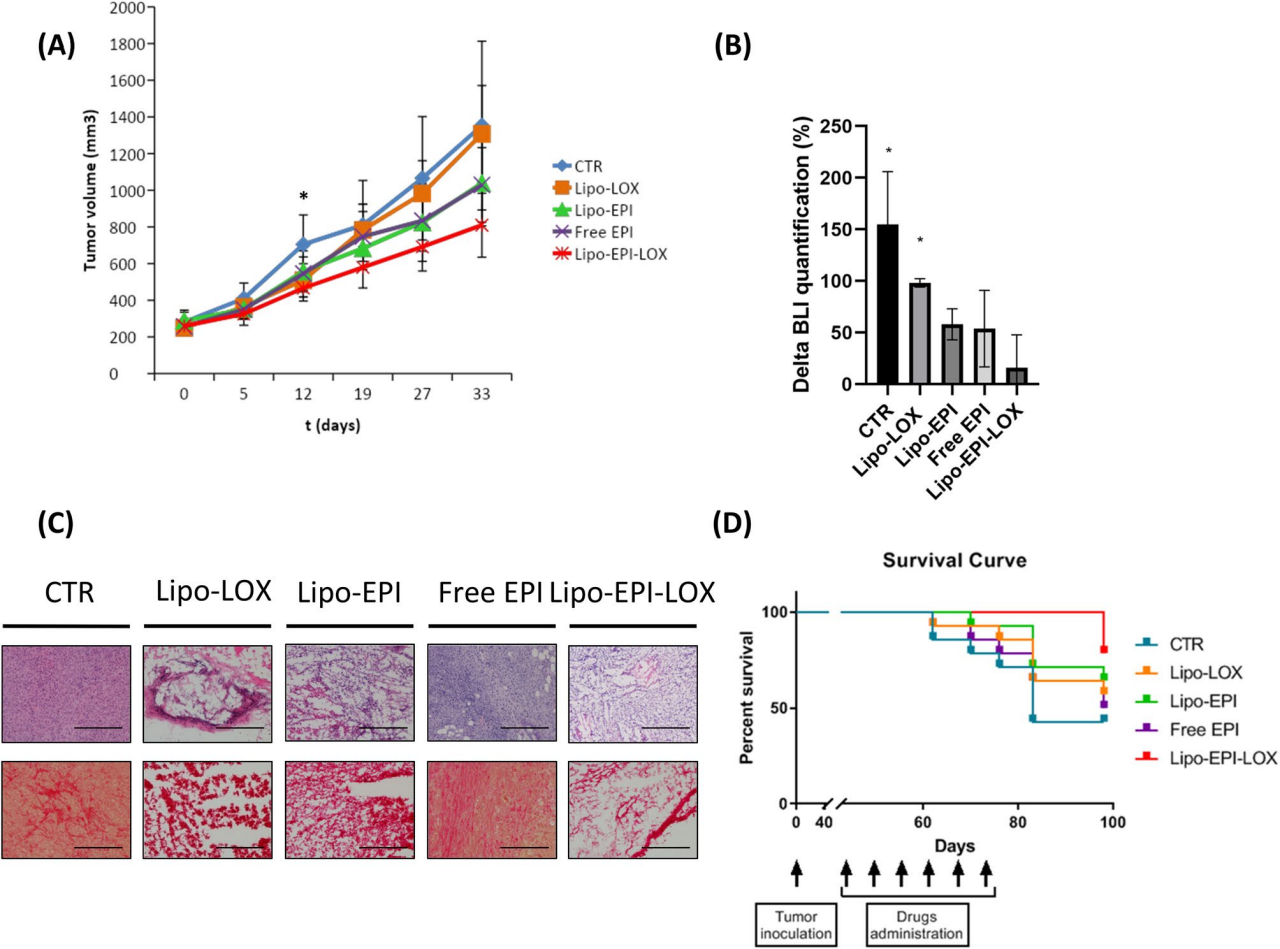
(**A**) Tumor growth curves of orthotopic xenograft mouse model of human TNBC. Five group mice (*n* = 10 each group) treated with empty liposome (CTR), Lipo-LOX, LIPO-EPI, free EPI and Lipo-EPI-LOX. (**B**) Delta quantification of TNBC bioluminescent signal after 4 weeks of treatment. (**C**) Representative example of H&E stained sections of explanted tumors 55 days post treatment. Control: markedly atypical and pleomorphic cells (5% necrotic tumor cells). Scale bar is 400 µm. Lipo-LOX: fibrotic tissue with atypical cells and neoplastic crown (25% necrotic tumor cells). Lipo-EPI: Neoplastic cells and necrotic bands (50% necrotic tumor cells). Free EPI: tumor cells infiltrating adipose tissue with minimal necrotic features e limited inflammation (20% necrotic tumor cells). Lipo-EPI-LOX : tumor cells infiltrating adipose tissue with artifacts (60% necrotic tumor cells). Representative example of collagen stained sections of explanted tumors 55 days post treatment. Control: collagen fibers linearization ˃ 200 µm, Lipo-LOX collagen fibers linearization ˂ 200 µm, Lipo-EPI collagen fibers linearization ˃ 200 µm, free EPI collagen fibers linearization ˃ 200 µm, Lipo-EPI-LOX collagen fibers linearization ˂ 200 µm. Scale bar is 400 µm. (**D**) Kaplan–Meier curve indicating survival in tumor-bearing mice after tumor induction in the listed treatment groups (Control, Lipo-LOX, LIPO-EPI, free EPI and Lipo-EPI-LOX).

### In vivo biodistribution and tumor targeting of engineered-anti-LOX liposomes

A critical directive in drug delivery is the selective delivery of cytotoxic compounds to the targeted site while avoiding healthy tissues. The biodistribution of EPI release from Lipo-EPI and Lipo-EPI-LOX was evaluated using an orthotopic xenograft mouse model of human TNBC. MDA-MB-231 were modified to express firefly luciferase and subcutaneously injected into the mammary fat pad of NU/NU nude mice. The tumor-bearing mice were split into two groups for intravenous treatment with Lipo-EPI or Lipo-EPI-LOX. Biodistribution analysis was assessed at 0, 1, 2, and 24 h using bioluminescent imaging (BLI) and fluorescence imaging to determine the distribution of tumor cells and EPI, respectively (Fig. 3A). The signal visible at 0 h represented the autofluorescence of mice in the 500–550 nm region. At 1 h, visible signals from EPI were observed in tumors (outlined in red after confirmation of location with BLI) of mice treated with Lipo-EPI-LOX and persisted until 24 h (Fig. 3A). In both groups, EPI signals in the abdomen and thoracic area were observed at 1 and 2 h but dissipated at 24 h. The mice were sacrificed at 24 h, at which point their organs were collected and they were imaged for EPI (Fig. 3B). Ex vivo imaging of organs showed that mice treated with Lipo-EPI-LOX exhibited more EPI accumulation in tumors and less accumulation in the liver and lungs compared to mice treated with Lipo-EPI. Quantification of longitudinal imaging in Fig. 3A confirmed that mice treated with Lipo-EPI-LOX displayed higher levels of EPI at 1 and 24 h in comparison to mice treated with Lipo-EPI (Fig. 3C). Furthermore, quantification of EPI in organs revealed significantly higher accumulation of EPI in tumors and significantly less accumulation in livers of mice treated with Lipo-EPI-LOX (Fig. 3D). Taken together, these data suggest that Lipo-EPI-LOX formulation delivered Epirubicin to TNBC tumors more efficiently than Liposome without anti-LOX surface-bound.

### Treatment with Lipo-EPI-LOX slowed the mammary progression and prolonged the survival of MDA-MB-231 murine xenografts

The therapeutic potential of Lipo-EPI-LOX was explored using the same TNBC tumor model described above. When tumors reached a mean volume of 250–300 mm^3^, mice were divided into groups and intravenously administered with a weekly treatment of empty liposome (i.e., control (CTR)), Lipo-LOX, Lipo-EPI, Free-EPI, and Lipo-EPI-LOX. In vivo analysis was designed to be a near-patient treatment regimen. Mice were treated for five weeks and sacrificed upon reaching a mean tumor volume of 2000 mm^3^. Weekly tumor volume measurements revealed that treatment with Lipo-EPI-LOX yielded significant inhibition of TNBC growth compared to all treatment groups at 12 days (Fig. 4A). In particular, Lipo-EPI-LOX displayed a substantial reduction (810 mm^3^ ± 174 mm^3^) in tumor growth compared to Lipo-EPI (1039 mm^3^ ± 233 mm^3^) and free EPI (1029 mm^3^ ± 203 mm^3^). Lipo-EPI and EPI were consistent with clinical observations where conventional anthracycline-based regimens and liposomal anthracycline exhibited equivalent antitumor efficacy^38^. Treatment with Lipo-EPI-LOX resulted in significant (*p* < 0.05) tumor growth inhibition beginning at day 12 and displayed a similar trend of significance from day 19 to day 33 in comparison to other treatments. Furthermore, the impact on tumor growth was validated by performing bioluminescence imaging (BLI) of TNBC tumors at day 0 and day 33 (Fig. 4B, Supplementary Fig. 5). This was performed to support the caliper measurements since the bioluminescent signal obtained from BLI is directly related to the viability of cells and it could serve as a metric to evaluate the impact of therapy^39^. The BLI quantification percentage (D0 vs D33) was found to be statistically significant in CTR (*p* = 0.01) and Lipo-LOX (*p* = 0.01) groups, while free EPI (*p* = 0.24), Lipo-EPI (*p* = 0.09) and Lipo-EPI-LOX (*p* = 0.35) did not result in statistical significance. Notably, the lowest BLI quantification percentage was observed with the Lipo-EPI-LOX treatment group. As in clinical practice, these experiments confirmed that anthracycline-based regimens can slow down mammary cancer progression and demonstrated that LIPO-EPI-LOX treatment provides superior antitumor efficacy among all treatment groups. The decreased viability detected by BLI analysis was further confirmed by hematoxylin and eosin (H&E) staining of tumor sections and they were analyzed for the percentage of necrotic cells. In particular, necrotic tumor cells were 5% in the control group, 25% in Lipo-LOX, 50% in Lipo-EPI, 20% in free EPI, and 60% in Lipo-EPI-LOX treated tumors (Fig. 4C). These experiments confirmed the superior ability of Lipo-EPI-LOX in inducing tumor necrosis compared to other treatment conditions. Moreover, since previous works^4,40^ have demonstrated that the progressive linearization of collagen fibers adjacent to tumor lesions is linked to collagen crosslinking, ECM stiffening, and increased focal adhesions, we investigated the collagen of explanted tumors. The analysis showed higher collagen linearization in CTR, Free EPI, and LIPO-EPI while a decrease in collagen stiffness and linearization was observed with LIPO-LOX and LIPO-EPI-LOX (Fig. 4C). Reducing LOX-mediated collagen crosslinking decreased focal adhesions and PI3K activity, hampered malignancy, and lowered tumor incidence^4^. Thus, these results support the ability of Lipo-EPI-LOX to reduce tumor progression among the tested treatment groups.

**Figure 5:**
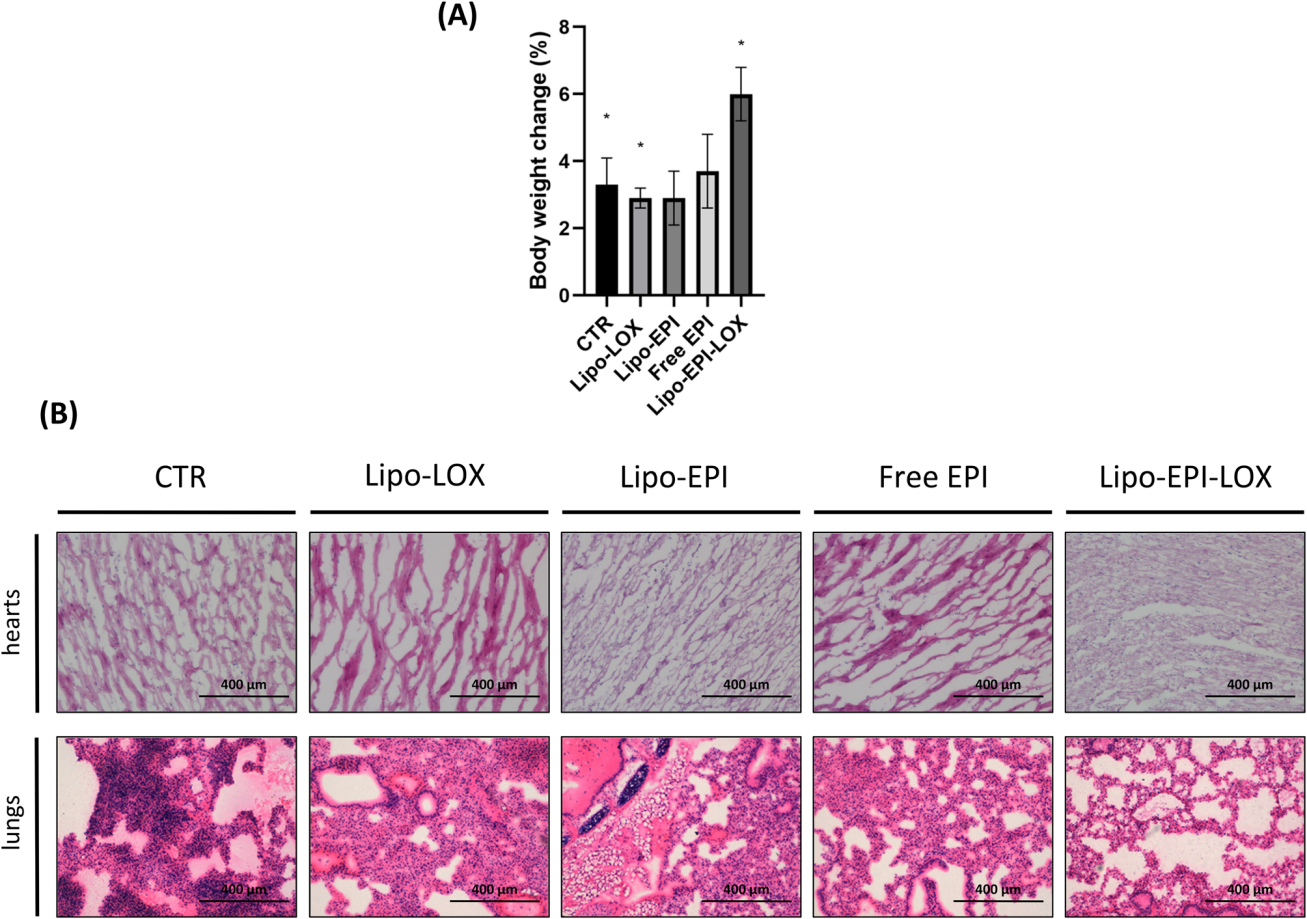
(**A**) body weight change percentage of mice at the end of the study. (**B**) Representative example of H&E stained sections of explanted hearts 55 days post treatment. Control: normal cardiac tissue. Lipo-LOX: no increase of inflammatory infiltrate. Lipo-EPI: minimal focal increase of inflammatory infiltrate (granulocytes and lymphocytes), artifacts. Free EPI: increased cellularity due to possible inflammatory infiltrate. Lipo-EPI-LOX : no significant increment of lymphoid infiltrate, artifacts. Scale bar is 400 µm. Representative example of H&E stained sections of explanted lungs 55 days post treatment. Control: phlogosis 70%. Lipo-LOX: phlogosis 30%. Lipo-EPI: phlogosis 15%. Free EPI: phlogosis 15%. Lipo-EPI-LOX : phlogosis 5%. Scale bar is 400 µm.

Following the establishment of Lipo-EPI-LOX antitumor activity, we investigated the impact of therapy on overall survival (Fig. 4D). Following the conclusion of the survival study (98 days post-inoculation), we observed that Lipo-EPI-LOX treatment exhibited > 75% survival, whereas control and free-EPI experienced survival rates < 50% and Lipo-LOX and Lipo-EPI exhibited < 65% survival rates. Taken together, Lipo-EPI-LOX led to a significant survival advantage (*p* = 0.032) relative to all groups. Besides, Lipo-LOX demonstrated prolonged survival when compared to an anthracycline regimen, but not to Lipo-EPI treatment. This confirms a potential synergistic effect mediated by both anti-LOX and EPI when administered via the same formulation. These findings provide a proof-of-concept assessment of Lipo-EPI-LOX as a viable therapeutic option for TNBC cancer patients.

### Safety of Lipo-EPI-LOX treatment on MDA-MB-231 murine xenografts

A major issue of chemotherapy is associated with systemic toxicity. Then, we analyzed the in vivo safety profile of LIPO-EPI-LOX by assessing the total change in body weight (Fig. 5A) and cardiotoxicity (Fig. 5B). Abnormal alterations in body weight can be used as a surrogate of the overall health status and tolerability of systemically administered substances^41^. Percent change in body weight was calculated by comparing weights at the start and end of therapy and resulted in the following increases: 3.3% in CTR (*p* = 0.05), 2.9% in Lipo-LOX (*p* = 0.008), 2.9% in Lipo-EPI (*p* = 0.06), 3.7% in Free EPI (*p* = 0.10) and 6.0% in Lipo-EPI-LOX (*p* = 0.004). In this regard, while mice of CTR, Lipo-LOX, Free EPI, and Lipo-EPI conditions experienced issues in feeding and drinking during disease progression this was slightly observed in the LIPO-EPI-LOX treatment group. In summary, these results demonstrate that Lipo-EPI-LOX treatment was the best tolerated therapy.

Anthracycline-based regimens have a well-documented history of cardiotoxicity^42^. For this reason, we evaluated the impact of our therapy on the architecture of the heart. Analysis of H&E hearts confirmed the safety of Lipo-EPI-LOX in preventing organ-related toxicity (Fig. 5B). Myocardial tissues treated with Free EPI and Lipo-EPI showed an increment of cellularity due to inflammatory infiltration (granulocytes and lymphocytes). Conversely, myocardial tissues from Lipo-LOX and Lipo-EPI-LOX groups did not show a significant increase in the inflammatory infiltrate. These results indicate that Lipo-EPI-LOX did not trigger a significant adaptive immune response in hearts against lipid-vesicles and suggests its ability to avoid significant cardiotoxicity in vivo. Moreover, in order to assess the safety profile of LIPO-EPI-LOX in other well-perfused organs, the explanted lungs architecture was investigated.

In this regard, 70% of phlogosis was detected in the control group, 30% in Lipo-LOX, 15% in Lipo-EPI, 15% for free EPI, and 5% for Lipo-EPI-LOX (Fig. 4C). Thus these results provide further evidence that LIPO-EPI-LOX was the best tolerated therapy.

Taken together, these results indicate that the Lipo-EPI-LOX treatment group led to better overall performance when compared to the other treatment groups.

## Discussion

In summary, we demonstrated the efficacy of LOX-engineered lipid vesicles loaded with EPI for the treatment of TNBC. We proved that a lipid-based nanocarrier targeting LOX and loaded with anthracycline is feasible and it could successfully provide significant therapeutic advantages (i.e. increased tumor growth inhibition and reduced toxicity). In vitro experiments showcased superior internalization and cytotoxic activity for Lipo-EPI-LOX compared to all the tested formulations. Also, in vivo experiments revealed Lipo-EPI-LOX exhibited higher therapeutic activity enabling a significant increase in the percentage of mice survival. When compared to current clinical standards, Lipo-EPI-LOX further demonstrated a reduction in systemic toxicity, solidifying its potential as a viable therapeutic strategy for TNBC and as an ECM-targeting formulation.

## Methods

### Fabrication and physical characterization of LOX engineered lipid vesicles

Lipid vesicles were prepared by the well-established TLE procedure. DSPC, cholesterol, DSPE-PEG(2000), and DSPE-PEG(2000)-succinyl (Avanti Polar Lipid) were dissolved in chloroform with a final lipid concentration of 20 mg/mL. The molar ratio of DSPC/cholesterol/DSPE-PEG(2000)/DSPE-PEG(2000)-succinyl was 60:30:5:5. The solvent was evaporated through a rotary evaporator (Buchi Labortechnik AG, Switzerland) for 20 min at 45 °C to form a thin lipid film. The film was hydrated with sterile water to assemble lipid vesicles (CTR). Then the solution was heated at 45 °C for three minutes and mixed with a vortex for three minutes, repeated for a total of three times. Lipid suspension was forced ten times through 2 polycarbonate filters (200 nm; GE Osmonics Labstore, Minnetonka, MN) under nitrogen gas pressure at 45 °C. Anti-LOX (MW 36 KDa, 100 mg/mL), was then added to this mixture, and the suspension was incubated overnight at 4 °C with gentle stirring (with different anti-LOX/lipid molar ratio, 1:1000, 1:500, 1:300). Liposome purification was performed by 1 h dialysis through 1000 KDa membranes (Spectrum Laboratories, Inc.) and then samples were stored at 4 °C. Physical characterization was carried out with a Nanosizer ZS (Malvern Instruments, Malvern, Worcestershire, UK). LOX conjugation was validated by flow cytometry analysis. Briefly, a secondary antibody (FITC-labeled, Alexa fluo 488) was incubated with liposomes and LOX-functionalized liposomes in MES buffer solution (1:1000 dilution, pH 7.5) for 1 h at room temperature. After 1 h dialysis through 1000 KDa membranes, the FITC signal was detected by flow cytometry. To further evaluate liposomes anti-LOX coupling Fourier Transform Infrared spectroscopy (FTIR) analysis in attenuated total reflection using a single reflection diamond element was performed. For the study, the FTIR spectrometer Nicolet was used. Lipid vesicles drug loading (Lipo-EPI and Lipo-EPI-LOX) was obtained through passive loading techniques. Lipid films were hydrated with a 33 mg/mL solution of ammonium chloride and dialyzed for 24 h in a 0.9% solution of sodium chloride to remove not encapsulated ammonium chloride. Particles were then incubated with epirubicin hydrochloride solution (epirubicin/lipid molar ratio 1:4) for 2 h at 45 degrees C. For drug encapsulation efficiency expressed in percentage, the concentration of epirubicin was determined in the lysed liposomes and the supernatant at 495 nm using a plate reader (BioTek, Synergy H4 hybrid reader) and analyzed based on a standard curve for epirubicin.

More details are available in the supplementary information.

### Theoretical calculations of lipo-LOX surface coupling

For anti-LOX quantification, a solution of 1 µg/ml of liposomes conjugated with anti-LOX was incubated with 2 µg/ml of LOX secondary antibody. Fluorescence obtained from Lipo or Lipo-EPI was subtracted as background. Results were interpolated in a LOX calibration curve previously prepared. Moreover, an indirect quantification of anti-LOX coupling to liposomes was performed with the analysis of supernatants. Finally, to corroborate the results previously obtained, the Bradford microassay (Quick Start Bradford Protein assay, Bio-Rad Laboratories, Los Angeles, CA) was used to quantify the amount of antibody conjugated to the liposome. A spectrophotometer was used to analyze the samples at 595 nm. The quantification was performed using a standard calibration curve obtained using bovine serum albumin. Anti-LOX concentration was expressed as the average between fluorescence and protein analysis results and converted to µg of antibody per 100 µg of polymer liposomes.

### Cells seeding and culturing

All the study was performed with the use of human breast cancer cell line MDA-MB-231 purchased from the American Type Culture Collection (Rockville, Maryland, USA). Cells were maintained in Dulbecco’s modified Eagle’s medium (DMEM) supplemented with 10% fetal bovine serum, 1% penicillin–streptomycin, and 1% glutamine (PAA, Piscataway, NJ, USA) at 37 °C in a 5% CO_2_ atmosphere. For standard cultures, 2.5 × 10^6^ cells were seeded and maintained as a monolayer in 75-cm^2^ flasks. Passages were performed according to the manufacturer's instructions. For tridimensional cultures, 5 × 10^6^ cells were cultured for 7 days in scaffolds synthesized as previously described^43^, the medium was replaced daily. All the experiments were conducted using low-passage MDA-MB-231 cell line and in active proliferation.

### Validation of lipo-LOX activity

To confirm the activity of LOX antibody after the liposome functionalization, secreted LOX protein was quantified in the cells culture medium at different time points. Briefly, MDA-MB-231 cultured both in standard monolayer and tridimensional culture were exposed to Lipo, EPI, Lipo-EPI, and Lipo-EPI-LOX at the concentration of the human plasma peak as specified in the drug testing section. Culture medium was collected at 6, 24, and 48 h respectively. The medium was centrifuged at 15,000 rpm for 20 min at 4 °C and the pellet was discarded to eliminate cell debris. The protein contents were determined using a BCA protein assay kit (Pierce BCA Protein Assay Kit, Thermo Scientific). An equal amount of protein from each sample was separated on Criterion Precast Gel Tris–HCl (Biorad, Hercules, CA, USA) and transferred to polyvinylidene fluoride membranes (Millipore Corporation)^44^. The membranes were blocked for 2 h with a solution containing 5% fat-free milk PBS with 0.1% Tween 20 (Sigma-Aldrich) at room temperature and incubated overnight at 4 °C with anti-LOX antibody (1:1000 ab31238, Abcam, Cambridge, UK). The membranes were then washed and incubated for 1 h at room temperature with horseradish peroxidase-conjugated secondary antibody following the protocol previously reported^45^.

### Microscopy analysis

To confirm the EPI delivery ability of the Lipo-LOX formulation, the confocal analysis was performed on 3D culture. The culture was exposed to Lipo-EPI and Lipo-EPI-LOX for 72 h, then the cells were washed 3 times with 1% PBS, fixed with 4% paraformaldehyde for 20 min at room temperature, and stained with DAPI (1:1000, Life Technologies, Carlsbad, CA, USA) and Phalloidin (1:40 Alexa Fluor 488 phalloidin, Life Technologies, Carlsbad, CA, USA). Images were acquired with an A1 laser confocal microscope (Nikon Corporation, Tokyo, Japan), and analyzed with the NIS Elements software (Nikon Corporation, Tokyo, Japan).

### In vitro drug testing

Cells were cultured in monolayer cultures or in 3D scaffolds for 24 h before exposure to drugs. Drug regimens were selected according to the plasma peak concentration of epirubicin from pharmacokinetic clinical data, 3.4 µg/ml^46^ (which correspond for lipo-EPI and lipo-EPI-LOX to 110 µg of lipid component per ml). For lipo-LOX and lipo-EPI-LOX treatment groups, the same concentration of theoretical LOX was used (0,11 µg of anti-LOX per ml). For lipo and lipo-LOX treatment groups, the same concentration of lipid vesicles was used (110 µg/ml). Cell viability percentage was assessed by 3-(4,5-Dimethylthiazol-2-yl)-2,5-Diphenyltetrazolium Bromide (MMT) assay (Sigma Aldrich, St. Louis, MO, USA) after 72-h of drug exposure as previously reported^47,48^. The IC_50_ values were calculated from the non-linear regression of the dose-log response curves. Experiments were performed in triplicate.

### In vivo study

All animal experiments were performed in accordance with the guidelines of the Animal Welfare Act and the Guide for the Care and Use of Laboratory Animals approved by the Institutional Animal Care and Use Committee (IACUC) of the Houston Methodist Research Institute (HMRI) protocol number AUP-0614-0033. The study was carried out in compliance with the ARRIVE guidelines.

### Orthotopic xenograft

MDA-MB-231 modified for luciferase expression (2 × 10^6^ cells/100 µl matrigel) were orthotopically injected into the right mammary fat pad of female immunodeficient NU/NU nude mice [NU-Foxn1nu] 8–10 weeks old (Charles River Laboratories, Wilmington, MA, USA). NU/NU nude mice were housed five per cage at the HMRI animal facility, they were maintained under pathogen-free conditions and on a low-fluorescence diet according to the National Institutes of Health guidelines.

### Tumor growth and body weight measurements

Tumor size and body weight were measured with a caliper and a digital balance once a week. Tumor volume was calculated according to the formula (*W *×* W *×* L*)/2, where *W* and *L* represent the width and the length of the tumor, respectively.

### In vivo drug testing

Once the tumors reached a 270 mm^3^ median volume NU/NU nude mice were sorted into groups for treatment with Lipo-LOX, Lipo-EPI, free EPI, Lipo-EPI-LOX, and CTR. Mice bearing MDA-MB-231 xenografts received weekly i.v. injections of drugs 3,24 mg/Kg (*n* = 10 per cohort) for five weeks. Dosages were selected according to the human plasma peak of epirubicin from pharmacokinetic clinical data and converted to mice equivalent surface area^49^. For the Lipo-LOX group, the equivalent concentration of liposomes used for the above groups was administered.

### Survival study

Mice (*n* = 10 per cohort) were sacrificed when the tumor volume reached 2000 mm^3^ or when showing signs of morbidity. Data were analyzed using the Kaplan–Meier method. Comparisons among different treatment groups were performed using the log-rank test for trend and considered significant with a *p*-value less than 0.05.

### Biodistribution of EPI in MDA-MB-231 tumors

Nu/Nu mice were implanted with MDA-MB-231 tumors as described above. When tumors reached a sufficient size, mice were imaged for bioluminescence and fluorescence to collect background signals. Imaging was performed using an IVIS Spectrum housed within HMRI’s Translation Imaging—Preclinical Imaging (Small Animal) Core, as previously described^39,50,51^. Here, we used bioluminescence imaging to determine the location of tumors using the same procedure discussed above. Fluorescence imaging was done by collecting images using 500/620 (excitation/emission) filters to follow the distribution of EPI. After imaging background signal (t = 0 h), mice were separated into groups for treatment with Lipo-EPI (*n* = 2) or Lipo-EPI-LOX (*n* = 3). At 1, 2, and 24 h, mice were imaged for bioluminescence and fluorescence. Mice were sacrificed after 24 h and their organs were imaged using the same settings. Images and quantification of EPI distribution were analyzed using Living Image 4.5, where bioluminescent images served to create tumor ROIs for each time point.

### Histology of organs and tumor tissues

Explanted mice hearts, lungs, and tumors were washed twice with PBS, embedded in a cryomold in O.C.T. (Tissue-Tek O.C.T. Compound, Sakura Finetek), and frozen at − 80 degrees. Ten μm -thick slides were obtained by cutting tissue blocks with a cryostat at − 20 degrees. The slides were then stored at − 20 degrees until analysis.

### Histological analysis

H&E staining was performed to evaluate the efficacy and safety of Lipo-EPI-LOX compared to the other treatment groups. Briefly, the organs and tumor slides were thawed, hydrated, and stained with H&E following the manufacturer’s instructions. The stained slides were analyzed with an optical Zeiss Axioskop microscope (Carl Zeiss, Gottïngen, Germany) equipped with a Polaroid camera as previously reported^43^.

### Collagen quantification

Tumors frozen 5-µm-thick slides were fixed for 5 min in formalin 10% solution, then washed and stained with Weigert’s hematoxylin. Then sections were stained with 0.1% (Direct Red 80) (Sigma Aldrich) picrosirius red in a saturated aqueous solution of picric acid. The stained slides were analyzed with the NIS Elements software (Nikon Corporation, Tokyo, Japan).

### Statistical analysis

Three independent replicates were performed for each experiment. Data are presented as mean ± SD, or mean ± SE, as stated, with n indicating the number of replicates. For in vitro and in vivo data, differences between groups were assessed by a two-tailed Student's t-test or analysis of variance (ANOVA) and accepted as significant at *p* < 0.05.

For EPI biodistribution studies, differences in EPI accumulation in tumors at various times and organs were assessed using multiple t-tests (one unpaired t-test per comparison) assuming samples with same scatter and accepted as significant at *p* < 0.05.

## Supplementary Information

Supplementary Information

## Data Availability

The datasets generated and/or analyzed during the current study are available from the corresponding author on reasonable request.
