# Supplementary material for: Lysyl oxidase engineered lipid nanovesicles for the treatment of triple negative breast cancer
Source: Sci Rep. 2021 Mar 3;11:5107. doi: 10.1038/s41598-021-84492-3 (PMC7930284; doi:10.1038/s41598-021-84492-3)
Supplement: Supplementary file 1 — Supplementary Information [file 41598_2021_84492_MOESM1_ESM.docx]

ARTICLE

**Lysyl oxidase engineered lipid nanovesicles for the treatment of triple negative breast cancer**

Alessandro De Vita^1*^, Chiara Liverani^1^, Roberto Molinaro^2,3,4^, Jonathan O. Martinez^3^, Kelly A. Hartman^3^, Chiara Spadazzi^1^, Giacomo Miserocchi^1^, Francesca Taraballi^3^, Michael Evangelopoulos^3^, Federica Pieri^5^, Alberto Bongiovanni^1^, Laura Mercatali^1^, Ennio Tasciotti^3,6§^ & Toni Ibrahim^1§^

^1^ Osteoncology and Rare Tumors Center, IRCCS Istituto Romagnolo per lo Studio dei Tumori (IRST) "Dino Amadori", Meldola, Italy

^2^ Department of Cardiovascular Medicine, Brigham and Women's Hospital, Harvard Medical School, Boston, MA, USA

^3^ Orthopedics and Sports Medicine, Houston Methodist Hospital, Houston, TX, USA

^4^ Business Development of Research, IRCCS San Raffaele Hospital, Milan, Italy

^5^ Pathology Unit, Morgagni-Pierantoni Hospital, Forlì, Italy

^6^ Biotechnology Program, San Raffaele University and IRCCS San Raffaele, Rome, Italy

**SUPPLEMENTARY INFORMATION**

**Fabrication and physical characterization of anti-LOX engineered lipid vesicles**

***Cytometry analysis***

For anti-LOX surface expression, anti-LOX-functionalized liposomes were prepared as previously described. Briefly, particles were added to 1% bovine serum albumin in phosphate buffered saline solution with a final concentration of 0.5 mM. Next, anti-LOX antibody (Abcam) was added at 2.5 μg/mL and allowed to incubate for 30 min. at 4 °C. Following incubation, particles were dialyzed using a Float-A-Lyzer G2 dialysis device (Spectrum Labs) with a 1000 kDa dialysis membrane for 1 h in Milli-Q water to remove unbound antibody. Following dialysis, particles were incubated with an AlexaFluor 488‐conjugated secondary antibody (goat anti-mouse IgG, Abcam) for 1 h at 4 °C and immediately followed by dialysis as previously described. Flow Cytometry was performed using a BD LSRFortessa cell analyzer with further analysis conducted using FlowJo X.

***Fourier Transform Infrared spectroscopy (FTIR) measurements***

Both Liposome and Lipo-LOX have been suspended at the concentration of 1 mg/ml in PBS. Fourier Transform Infrared spectroscopy was employed for the particles characterization, since it enabled the detection of the proteic part over the lipidic structure of the liposomes. Fourier Transform Infrared spectroscopy measurements in attenuated total reflection (ATR) were performed using a single reflection diamond element. The FTIR spectrometer Nicolet was employed under the following conditions: 2 cm^−1^ spectral resolution, 20 kHz scan speed, 1000 scan co-addition, and triangular apodization. 5 μl of each sample were deposited on the ATR plate and spectra were recorded after solvent evaporation to allow the formation of a hydrated lipophilic film. 5 ul of the buffer has been recorded to use as background. The ATR/FTIR spectra were reported after binomial smoothing (11 points) and the subtraction of background.

***Mechanical Testing***

As previously described^8^ an in-house built instrument prototype was developed by our group to apply a state of unconfined uniaxial compression to the collagen scaffolds. The applied stress (r, in kPa) could then be analyzed by dividing the applied force by the cross section of the sample. The applied force consisted of a pre-load of 1.41 kPa helded constantly for 30 s; the full load of 5.97 kPa was then constantly applied for 30 s. The stiffness (compressive modulus, in kPa) of the collagen scaffold cultured with MDA-MB-231 and exposed for 72h to all treatment groups was computed as the ratio between the increment of stress and increment of strain from the preload to the full load values. The samples were analyzed in air immediately after having been removed from the PBS where they were soaked. Five samples of each type were tested and each specimen was tested 10 times.


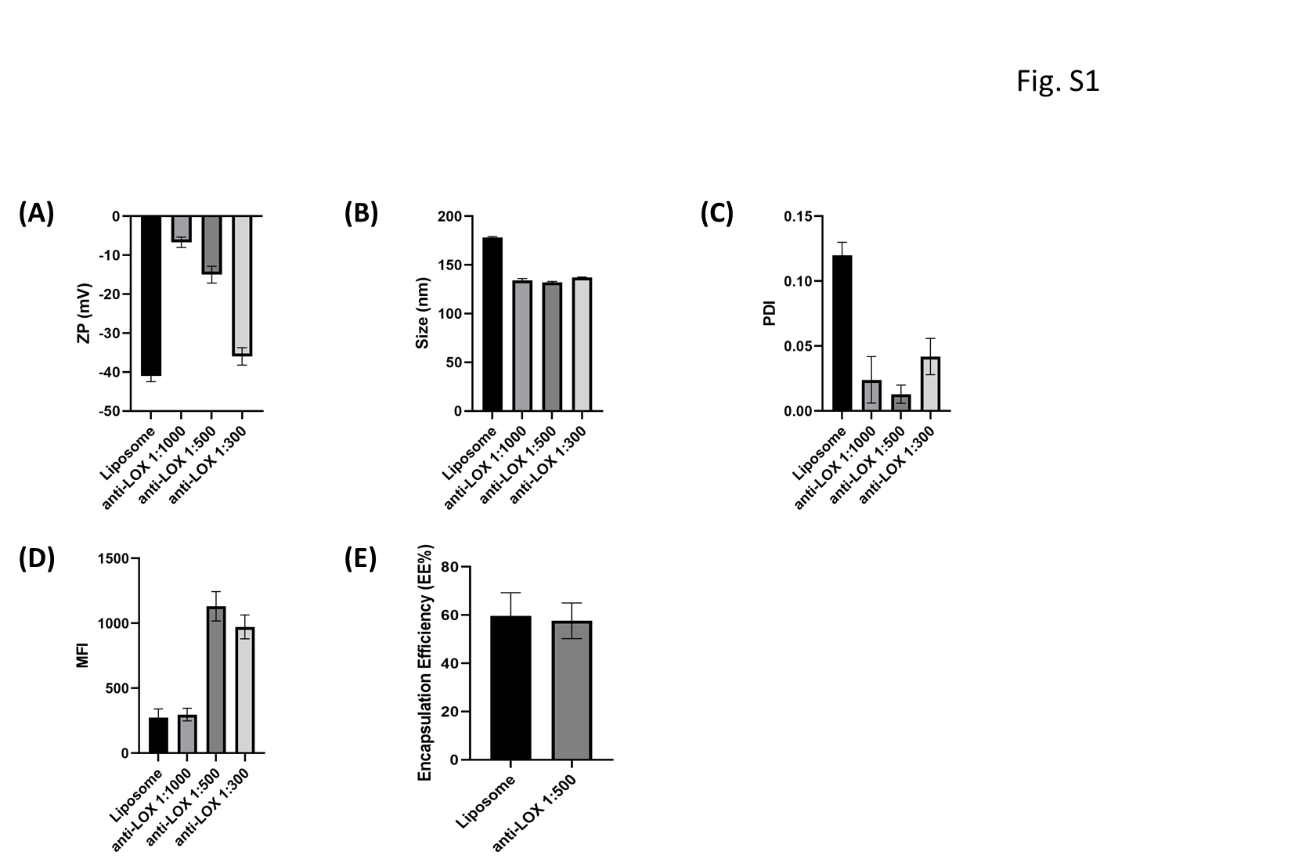


**Supplementary Figure 1**. Physicochemical characterization of PEGylated liposomes (liposomes) and LOX-conjugated PEGylated liposomes (LOX). Three different LOX-to-lipid ratio (1:000, 1:500, and 1:300) were tested. The presence of the LOX on the carrier's surface affected the surface charge, causing a significant reduction of the zeta potential (ZP) values (**A**). DLS analysis shows how the conjugation of LOX on the surface of liposomes brought to a collapse of the vesicular structure, as can be noted by the reduction of vesicle diameter (**B**), as well as a stabilization of the formulation, as indicated by the reduction of the PDI (**C**). Next, flow cytometry analysis was performed in order to evaluate the best LOX-to-lipid ratio in terms of LOX density on liposome surface (**D**). The analysis revealed that 1:500 LOX-to-lipid ratio permitted to obtain the highest density of LOX on the surface compared to 1:1000 and 1:300. Lastly, the encapsulation of epirubicin using the remote loading method revealed a loading of about 60% (**E**). Most importantly, compared to liposomes, the presence of LOX on the surface did not significantly affect the amount of drug encapsulated.


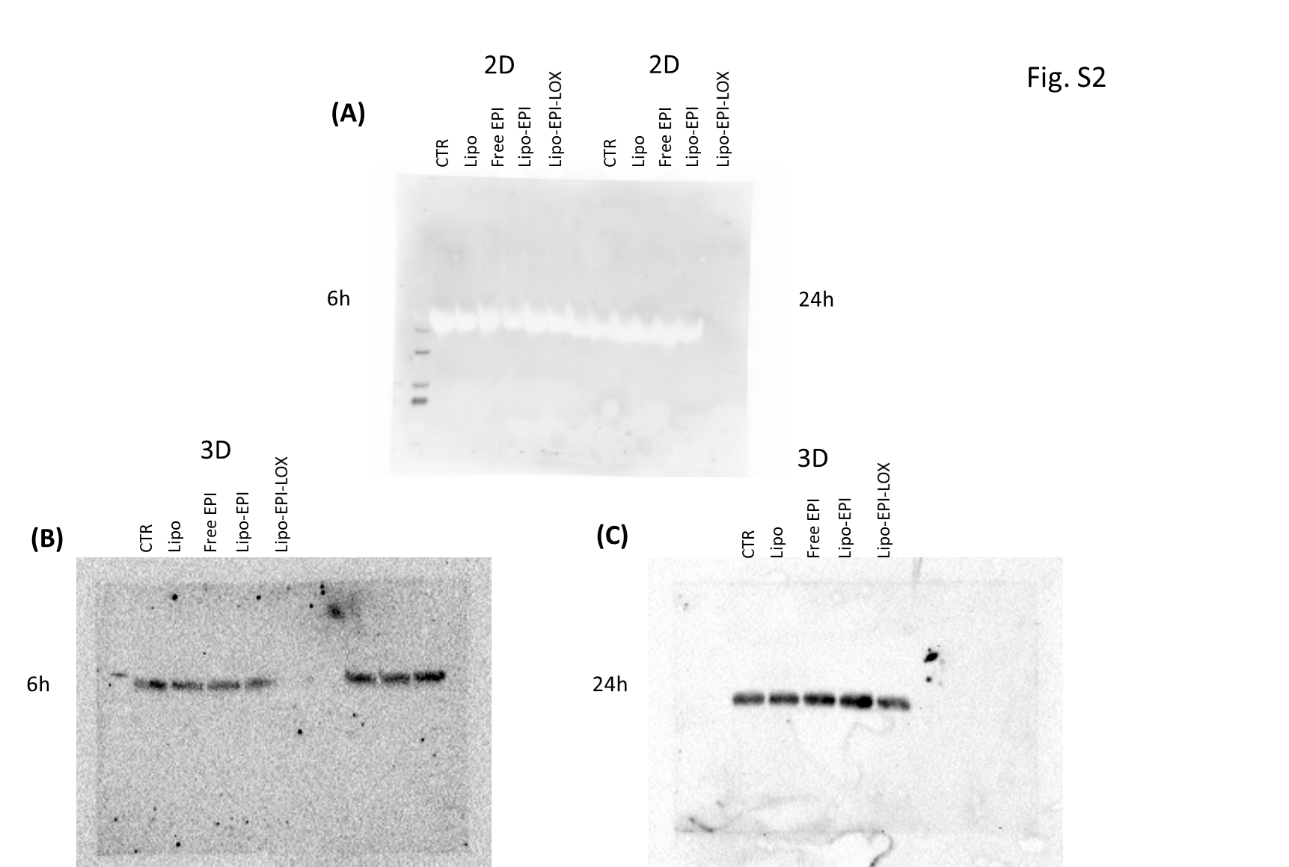


**Supplementary Figure 2**. (**A**) Protein analysis of secreted LOX in monolayer cultures not treated and treated with all formulations at 6h and 24h. (**B**,**C**) Protein analysis of secreted LOX in 3D cultures not treated and treated with all formulationsat at 6h (**B**) and 24h (**C**).

**
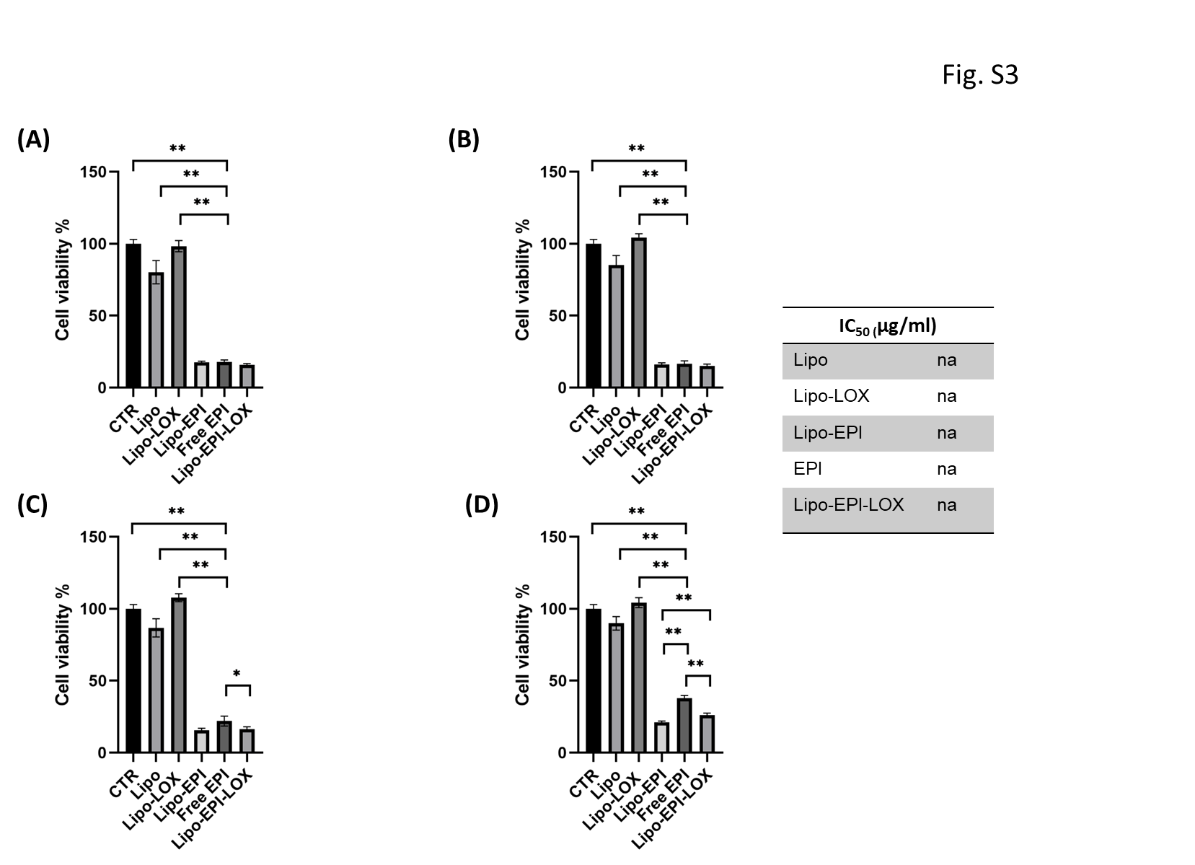
**

**Supplementary Figure 3**. Cell viability of tumor cells after treatment with all studied formulations in standard monolayer cultures. (**A**) epirubicin plasma peak concentration, (**B**) epirubicin half-plasma peak concentration, (**C**) epirubicin one-quarter plasma peak concentration, (**D**) epirubicin one-eight plasma peak concentration **p* < 0.05, ***p* < 0.01.


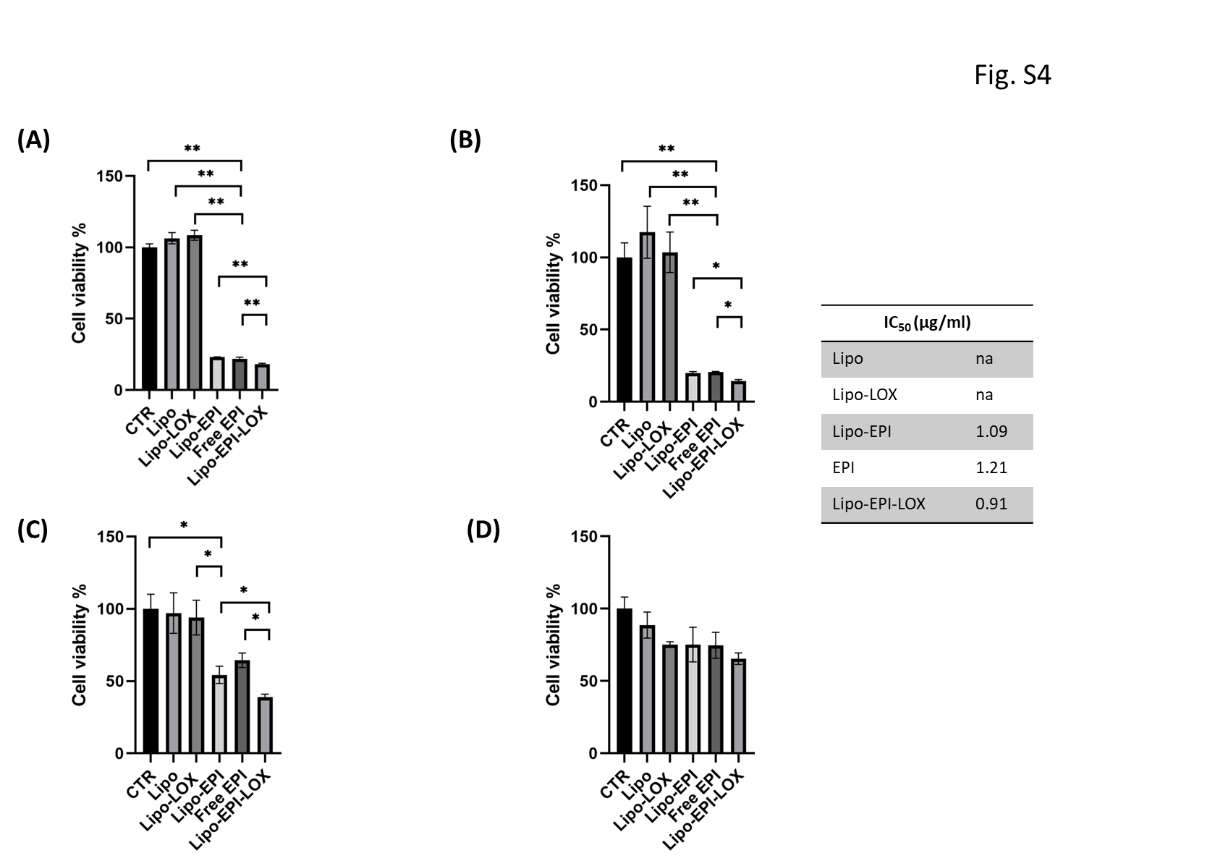


**Supplementary Figure 4**. Cell viability of tumor cells after treatment with all studied formulations in 3D cultures. (**A**) epirubicin plasma peak concentration, (**B**) epirubicin half-plasma peak concentration, (**C**) epirubicin one-quarter plasma peak concentration, (**D**) epirubicin one-eight plasma peak concentration **p* < 0.05, ***p* < 0.01.

**
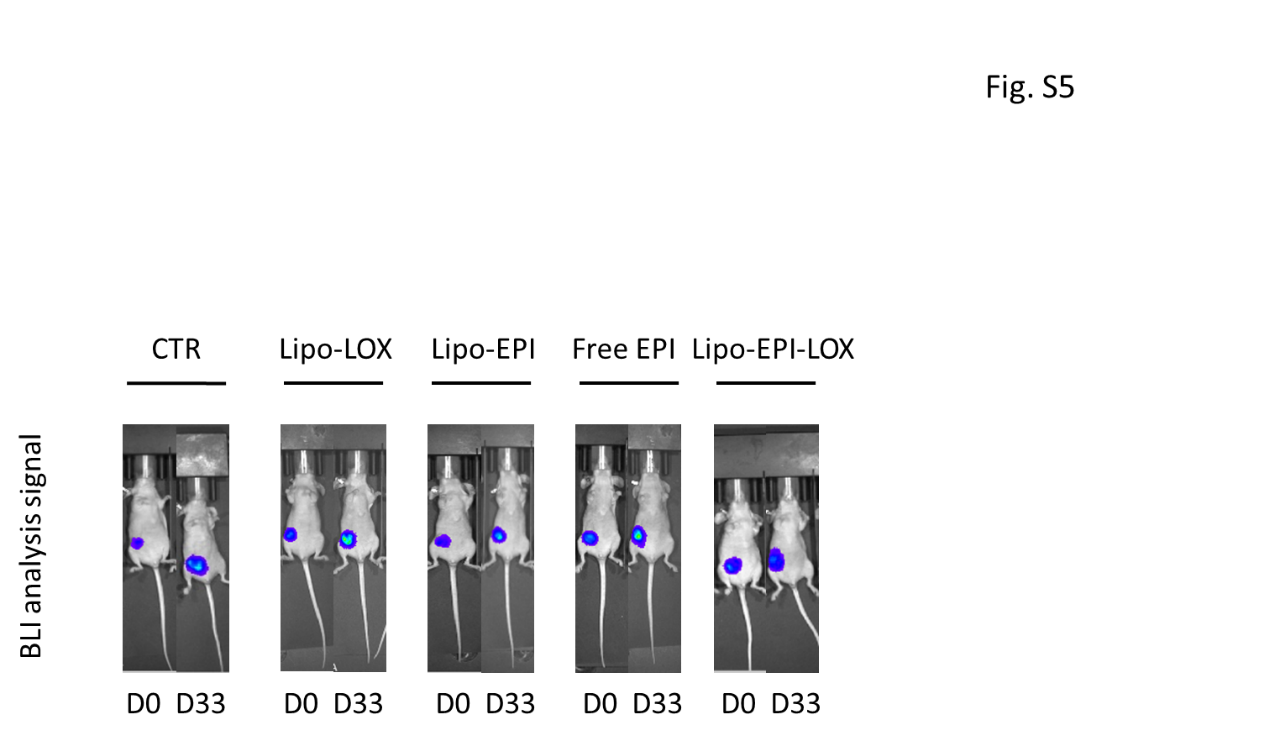
**

**Supplementary Figure 5**. Bioluminescence signal of TNBC in all treatments at starting treatments (D0) and after 4 weeks of treatments.

**
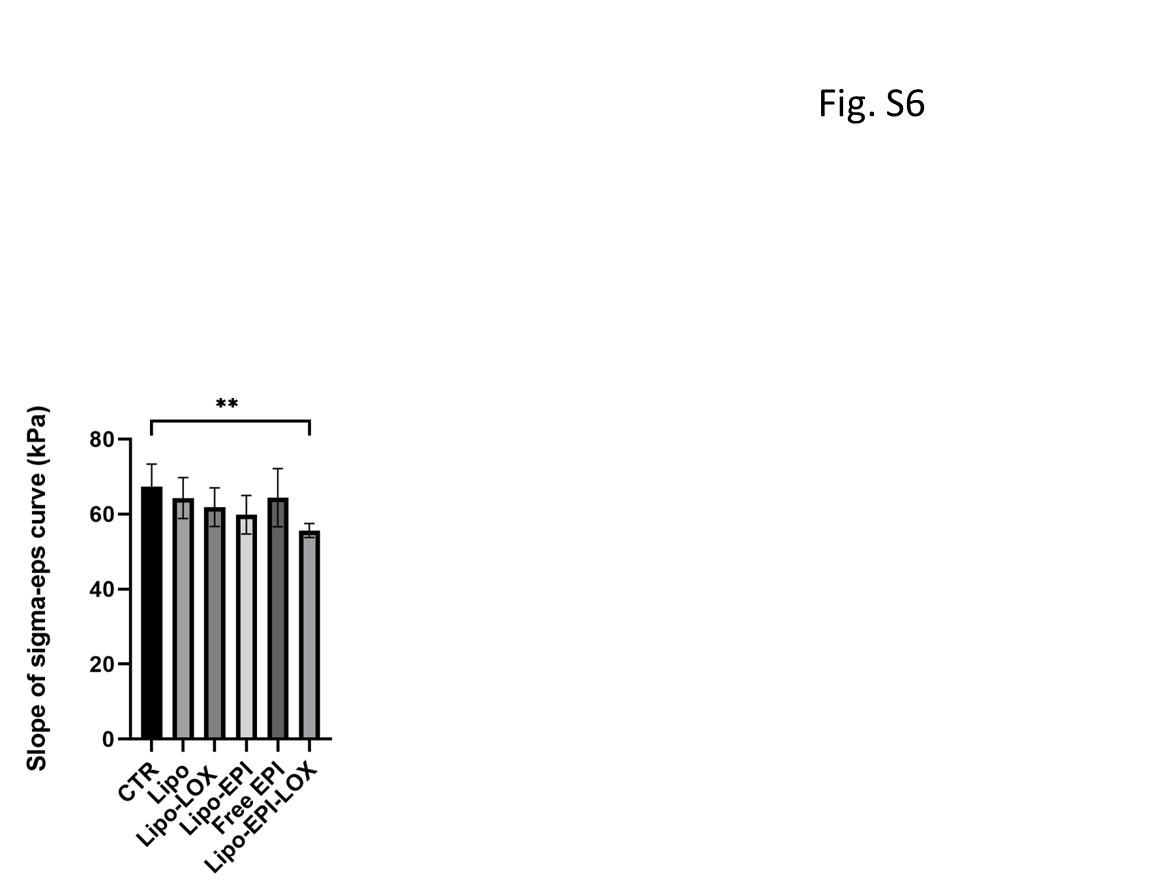
**

**Supplementary Figure 6**. Stiffness values for CTR, Lipo, Lipo-LOX, Lipo-EPI, Free EPI, Lipo-EPI-LOX scaffolds after 72 h of treatment, expressed as the slope of the sigma-epsilon curve (sigma-eps) (kPa).
